# Effect of Caffeine-Containing Beverage Consumption on Serum Alanine Aminotransferase Levels in Patients with Chronic Hepatitis C Virus Infection: A Hospital-Based Cohort Study

**DOI:** 10.1371/journal.pone.0083382

**Published:** 2013-12-11

**Authors:** Yachiyo Sasaki, Satoko Ohfuji, Wakaba Fukushima, Akihiro Tamori, Masaru Enomoto, Daiki Habu, Shuji Iwai, Sawako Uchida-Kobayashi, Hideki Fujii, Susumu Shiomi, Norifumi Kawada, Yoshio Hirota

**Affiliations:** 1 Department of Gerontological Nursing, Graduate School of Nursing, Osaka City University, Osaka, Japan; 2 Department of Public Health, Graduate School of Medicine, Osaka City University, Osaka, Japan; 3 Department of Hepatology, Graduate School of Medicine, Osaka City University, Osaka, Japan; 4 Department of Medical Nutrition, Graduate School of Human Life Science, Osaka City University, Osaka, Japan; 5 Department of Nuclear Medicine, Graduate School of Medicine, Osaka City University, Osaka, Japan; University of Modena & Reggio Emilia, Italy

## Abstract

**Introduction:**

To date, there have been no prospective studies examining the effect of coffee consumption on serum alanine aminotransferase (ALT) level among individuals infected with the hepatitis C virus (HCV). We conducted a hospital-based cohort study among patients with chronic HCV infection to assess an association between baseline coffee consumption and subsequent ALT levels for 12 months.

**Materials and Methods:**

From 1 August 2005 to 31 July 2006, total 376 HCV-RNA positive patients were recruited. A baseline questionnaire elicited information on the frequency of coffee consumption and other caffeine-containing beverages. ALT level as a study outcome was followed through the patients’ medical records during 12 months. The association between baseline beverage consumption and subsequent ALT levels was evaluated separately among patients with baseline ALT levels within normal range (≤45 IU/L) and among those with higher ALT levels (>45 IU/L).

**Results:**

Among 229 patients with baseline ALT levels within normal range, 186 (81%) retained normal ALT levels at 12 months after recruitment. Daily drinkers of filtered coffee were three times more likely to preserve a normal ALT level than non-drinkers (OR=2.74; *P*=0.037). However, decaffeinated coffee drinkers had a somewhat inverse effect for sustained normal ALT levels, with marginal significance (OR=0.26; *P*=0.076). In addition, among 147 patients with higher baseline ALT levels, 39 patients (27%) had ALT reductions of ≥20 IU/L at 12 months after recruitment. Daily drinkers of filtered coffee had a significantly increased OR for ALT reduction (OR=3.79; *P*=0.034). However, in decaffeinated coffee drinkers, OR could not be calculated because no patients had ALT reduction.

**Conclusion:**

Among patients with chronic HCV infection, daily consumption of filtered coffee may have a beneficial effect on the stabilization of ALT levels.

## Introduction

Hepatitis C virus (HCV) infection is a main cause of hepatocellular carcinoma. Approximately 150 million people are chronically infected with HCV worldwide [1] and about two million patients are located in Japan [2]. In the natural course of HCV infection, 20%–30% of patients develop liver cirrhosis in 10–20 years of follow-up, and 3%–4% of cirrhotic patients develop hepatocellular carcinoma in the first 4–5 years [3]. In particular, patients with higher alanine aminotransferase (ALT) levels have been reported to be at high risk for the development of hepatocellular carcinoma [4]. Therefore, identification of factors to control the ALT level is an important issue in patients with HCV.

To date, several epidemiological studies have reported the beneficial effects of coffee intake for hepatocellular carcinoma [5,6], liver cirrhosis [7-9] and serum liver enzymes [10-21]. However, most of the results about the association with lower ALT levels were from cross-sectional studies and were therefore concerned about reverse causality. Liver dysfunction is often accompanied by gastrointestinal disorders or impaired caffeine clearance [22], which may lead to reduced coffee consumption. Therefore, to elucidate the causal relationship, an assessment using a prospective study design was needed. To the best of our knowledge, there has been only one prospective cohort study on this topic [21]; that study targeted Japanese office workers who were free of liver dysfunction and showed that coffee consumption prevents ALT elevation. However, no prospective studies among patients with liver disease have investigated the association between coffee consumption and serum ALT level.

Thus, we successfully conducted a hospital-based cohort study to examine the association between baseline coffee consumption and subsequent serum ALT levels for 12 months among patients with chronic HCV infection. 

## Materials and Methods

### Study subjects

From 1 August 2005 to 31 July 2006 (recruitment period), all consecutive patients with chronic HCV infection who visited the Department of Hepatology at Osaka City University Hospital for clinical follow-up were invited to participate in the study. Eligible patients were those with a detectable HCV-RNA level at the time of recruitment. Exclusion criteria were as follows: patients with the other types of liver disease (e.g., co-infection with hepatitis B virus, autoimmune hepatitis, primary biliary cirrhosis, idiopathic portal hypertension, etc.); patients with previously diagnosed hepatocellular carcinoma; or patients who were receiving interferon (IFN) therapy at the time of recruitment.

The study protocol was approved by the ethics committee of the Osaka City University Graduate School of Medicine, and was performed in accordance with the Declaration of Helsinki. The study patients gave their written informed consent.

### Information collection at baseline

At the time of recruitment, each subject filled out a self-administered questionnaire. The questionnaire included items on demographic characteristics, past medical history (including surgery, blood transfusion and acupuncture before 1990, when HCV could be determined), potential behavior for relevant infection (e.g., needle/syringe sharing and tattoo), alcohol drinking, smoking, and consumption of coffee and other caffeine-containing beverages (e.g., green tea, black tea/oolong tea). Coffee consumption was separately asked by brewing method (filtered/ unfiltered/ decaffeinated) and the frequency of consumption was investigated on a daily, weekly, monthly scale or none. A question on other caffeine-containing beverages asked about the frequency using 8 levels (none, <1 cup/week, 1 cup/week, 2-3 cups/week, 4-6 cups/week, 1 cup/day, 2-3 cups/day and ≥4 cups/day). Duration of HCV infection was estimated if the date of surgery, blood transfusion, acupuncture, needle/syringe sharing or tattoo was known. The youngest age at the time of these procedures was adopted when the subject had undergone two or more procedures.

In addition, well-trained research nurses abstracted baseline laboratory data from the medical records of the study subjects. Laboratory data included serum ALT level (IU/L), platelet count (10×10^4^/μL), albumin level (g/dL) and total bilirubin level (mg/dL) etc.

### Follow-up survey

The study subjects were followed up for 12 months through their regular medical examinations, since most patients visited the hospital at 1- to 3-month intervals. From their medical records, we obtained the data on their ALT levels, at approximately 6 months and 12 months after recruitment as the study outcomes.

In addition, information on IFN therapy during the follow-up period was also abstracted, since the therapy would affect the study outcomes.

### Statistical analysis

The association between baseline beverage consumption and subsequent ALT levels was evaluated separately among patients with baseline ALT levels within normal range (≤45 IU/L) and among those with higher ALT levels (>45 IU/L), which was defined using the conventional cut-off value when the present study conducted. Patients who received IFN therapy during the follow-up period were excluded from analysis.

Baseline beverage consumption was categorized into three levels according to the distribution of consumption for all the study subjects. The category boundaries were drawn so as to make the size of the groups as similar as possible.

Among the patients with baseline ALT levels within normal range, the outcome was regarded as retaining normal ALT levels (≤45 IU/L) at each point of follow-up. If patients had abnormal ALT after 6 months and normal ALT after 12 months, they were classified as absence of outcome occurrence (retaining normal ALT levels) after 6 months and as presence of outcome after 12 months. Contrary to this, if patients had normal ALT after 6 months and abnormal ALT after 12 months, they were classified as presence of outcome occurrence after 6 months and as absence of outcome after 12 months.

 Among patients with higher baseline ALT levels, an ALT reduction of ≥10 IU/L, ≥20 IU/L or ≥30 IU/L was defined as the outcome. For example, in the analysis with ALT reduction of ≥10 IU/L as an outcome, if patients’ ALT levels were reduced by 15 IU/L after 6 months and 8 IU/L after 12 months, both of which compared with baseline ALT levels, they were classified as presence of outcome occurrence after 6 months and as absence of outcome after 12 months.

Logistic regression analysis was employed to estimate the odds ratios (ORs) and 95% confidence intervals (95% CIs) of baseline beverage consumption for outcomes. The trend of the association was evaluated by assigning ordinal scores to each level of the category. Variables which seemed to associate with baseline consumption of filtered coffee (*P*<0.05) or reported associations with ALT levels from previous studies [23-25] were considered to be potential confounders for adjustment. The chi-square test or Fisher’s exact test and the Kruskal-Wallis test were also used where appropriate. All *P* values were two-sided, and the level of significance was 5%. SAS version 9.1 (SAS Institute, Inc., Cary, NC) was used throughout the analysis.

## Results

Among 473 eligible patients, 61 patients started IFN therapy during the follow-up and were then excluded from analysis. In addition, follow-up data could not be obtained from 36 patients. Eventually, the data of 376 patients (229 with baseline ALT levels within normal range and 147 with higher ALT levels) were analyzed.

### Baseline characteristics of study patients

Table 1 shows the baseline characteristics of the study patients according to filtered coffee consumption. Among the 229 patients with normal ALT levels at baseline, higher filtered coffee consumption was significantly associated with younger age, higher proportion of current smokers and current alcohol drinkers. Even among the 147 patients with higher ALT levels at baseline, higher consumption of filtered coffee was likely to be current smokers, with marginal significance. Baseline laboratory data indicated that patients with advanced liver disease (represented by platelet count <10×10^4^/μl) did not frequently drink filtered coffee, both among patients with normal ALT levels and those with higher ALT levels at baseline.

**Table 1:** Baseline characteristics of study subjects according to filtered coffee consumption.

		Filtered coffee consumption			
Variable		Never	<1cup/day	≥1cup/day	p
Normal ALT (n=229)					
	No. of subjects	131	44	54	
	Sex, male %	36	32	41	0.632
	Age, years, mean±SD	68±11	62±10	62±11	0.0001
	BMI, kg/m^2^, mean±SD	22±3	22±3	22±3	0.218
	Smoking states, current %	15	11	24	0.008
	Alcohol drinking status, current %	20	34	35	0.017
	Diabetes mellitus, present %	8	7	4	0.710
	Laboratry data				
	ALT, IU/L, mean±SD	32±9	30±7	30±8	0.059
	Platelet count, <10×10^4^/μl %	13	2	6	0.064
	Albumin, <3.5g/dl %	6	5	2	0.532
	Total Bilirubin, ≥1.0mg/dl %	18	28	9	0.338
	Duration of HCV infection, years, mean±SD*	40±13	45±11	37±13	0.034
Higher ALT (n=147)					
	No. of subjects	89	28	30	
	Sex, male %	44	25	53	0.697
	Age, years, mean±SD	66±10	65±10	60±12	0.107
	BMI, kg/m^2^, mean±SD	23±3	23±3	22±3	0.904
	Smoking states, current %	13	11	30	0.057
	Alcohol drinking status, current %	18	25	30	0.379
	Diabetes mellitus, present %	17	7	27	0.443
	Laboratry data				
	ALT, IU/L, mean±SD	77±46	78±45	79±32	0.820
	Platelet count, <10×10^4^/μl %	37	25	10	0.004
	Albumin, <3.5g/dl %	15	0	7	0.058
	Total Bilirubin, ≥1.0mg/dl %	43	11	37	0.191
	Duration of HCV infection, years, mean±SD*	38±12	36±12	37±11	0.717

*P* values of characteristics between categories of filtered coffee consumption were calculated by analysis of chi-square test or Fisher's exact test and Kruskal-Wallis test.

SD, standard deviation; ALT, alanine aminotransferase; BMI, body mass index

^*^ 182 patients among the normal ALT group and 107 patients among the higher ALT group were analyzed; the duration of HCV infection could not be calculated in 87 patients due to lack of information.

We further analyzed the baseline characteristics of the study patients according to the consumption of other caffeine-containing beverages. As for unfiltered coffee, a similar relationship to the consumption of filtered coffee was observed. With regard to green tea (Table 2), higher green tea consumption was significantly related to female sex, older age, lower proportion of current smokers, and lower total bilirubin levels among the patients with normal ALT levels at baseline. Even among the patients with higher ALT levels at baseline, higher green tea consumption was associated with lower total bilirubin levels. No significant relationship was observed in decaffeinated coffee or black tea/oolong tea.

**Table 2:** Baseline characteristics of study subjects according to green tea consumption.

		Green tea consumption			
Variable		≤1 cup/week	2 cups/week-1cup/day	≥2 cups/day	p
Normal ALT (n=229)					
	No. of subjects	44	62	123	
	Sex, male %	48	40	30	0.027
	Age, years, mean±SD	66±12	62±12	67±9	0.016
	BMI, kg/m^2^, mean±SD	22±3	22±3	22±3	0.689
	Smoking states, current %	25	15	14	0.023
	Alcohol drinking status, current %	32	27	24	0.195
	Diabetes mellitus, present %	7	7	7	1.000
	Laboratory data				
	ALT, IU/L, mean±SD	30±8	31±8	32±9	0.318
	Platelet count, <10×10^4^/μl %	14	8	8	0.352
	Albumin, <3.5 g/dl %	7	6	3	0.486
	Total Bilirubin, ≥1.0 mg/dl %	25	25	11	0.016
	Duration of HCV infection, years, mean±SD*	41±12	37±11	43±13	0.013
Higher ALT (n=147)					
	No. of subjects	45	38	64	
	Sex, male %	44	53	34	0.240
	Age, years, mean±SD	63±11	63±12	66±10	0.130
	BMI, kg/m^2^, mean±SD	23±4	22±3	22±3	0.454
	Smoking states, current %	24	11	14	0.065
	Alcohol drinking status, current %	20	21	23	0.534
	Diabetes mellitus, present %	22	17	14	0.274
	Laboratory data				
	ALT, IU/L, mean±SD	84±49	71±27	77±46	0.391
	Platelet count, <10×10^4^/μl %	31	24	31	0.925
	Albumin, <3.5g/dl %	16	14	5	0.110
	Total Bilirubin, ≥1.0mg/dl %	47	37	27	0.030
	Duration of HCV infection, years, mean±SD*	38±12	35±12	39±12	0.280

*P* values of characteristics between categories of green tea consumption were calculated by analysis of chi-square test or Fisher's exact test and Kruskal-Wallis test.

SD, standard deviation; ALT, alanine aminotransferase; BMI, body mass index

^*^ 182 patients among the normal ALT group and 107 patients among the higher ALT group were analyzed; the duration of HCV infection could not be calculated in 87 patients due to lack of information.

Baseline characteristics of the study patients and beverages consumption were compared between patients with normal baseline ALT levels and those with higher levels according to baseline ALT levels (Table 3). Compared with patients who had normal baseline ALT levels, patients with higher baseline ALT levels had significantly higher proportions of diabetes mellitus, platelet count (<10×10^4^/μl), albumin (<3.5g/ dL), and total bilirubin (≥1.0 mg/dL), suggesting that patients with higher baseline ALT levels had more advanced liver disease. As for the beverages consumption, patients with normal baseline ALT levels had significantly higher consumption of green tea.

**Table 3:** Baseline characteristics of the study subjects and beverages consumption according to baseline ALT levels.

			Baseline ALT levels		
		Variable	Normal (≤45IU/L)	Higher (>45IU/L)	p
	No. of subjects		229	147	
	Sex, male %		36	42	0.250
	Age, years, mean±SD		65±11	65±11	0.382
	BMI, kg/m^2^, mean±SD		22±3	23±3	0.201
	Smoking states, current %		16	16	0.736
	Alcohol drinking status, current %		26	22	0.400
	Diabetes mellitus, present %		7	17	0.001
	Laboratry data				
	ALT, IU/L, mean±SD		31±8	78±43	<.0001
	Platelet count, <10×10^4^/μl %		9	29	<.0001
	Albumin, <3.5g/dl %		5	10	0.039
	Total Bilirubin, ≥1.0mg/dl %		18	35	0.0001
	Duration of HCV infection, years, mean±SD*		41±13	38±12	0.069
	Beverages consumption, %				
	Filtered coffee				
		Non-drinker	57	61	0.455
		<1cup/day	19	19	
		≥1cup/day	24	20	
	Unfiltered coffee				
		Non-drinker	53	56	0.509
		<1cup/day	17	18	
		≥1cup/day	30	27	
	Decaffeinated coffee				
		Non-drinker	96	99	0.137
		Drinker	4	1	
	Green tea				
		≤1cup/week	19	31	0.013
		2cups/week-1cup/day	27	26	
		≥2cups/day	54	44	
	Black tea /Oolong tea				
		Non-drinker	33	42	0.269
		<1cup/week-3cups/week	36	28	
		≥4cups/week	31	31	

*P* values of characteristics and beverages consumption between categories of baseline ALT levels were calculated by analysis of chi-square test or Fisher's exact test and Kruskal-Wallis test.

SD, standard deviation; ALT, alanine aminotransferase; BMI, body mass index

^*^ 182 patients among the normal ALT group and 107 patients among the higher ALT group were analyzed; the duration of HCV infection could not be calculated in 87 patients due to lack of information.

### Beverage consumption and retained normal ALT among patients with normal baseline ALT

During the 12 months of follow-up, those who retained normal ALT levels were 185 (81%) after 6 months and 186 (81%) after 12 months, respectively (Table 4). When logistic regression analysis was employed to estimate the effect of baseline beverage consumption for sustained normal ALT levels at each follow-up point, daily consumption of filtered coffee (≥1 cup/day) revealed significantly increased ORs for sustained normal ALT levels, both after 6 months (OR of daily drinker, 2.98; 95% CI: 1.06-8.34) and after 12 months (OR of daily drinker, 2.74; 95% CI: 1.00-7.54), with a significant dose-response relationship (*P* for trend = 0.037 for each). On the other hand, decaffeinated coffee drinkers showed decreased ORs for normal ALT levels during follow-up, which indicated a harmful effect for retaining normal ALT levels. In particular, for the outcome after 12 months, the decreased OR of decaffeinated coffee drinkers reached the level of marginal significance (OR of drinkers, 0.26; 95% CI: 0.06-1.15; *P*=0.076). In addition, higher consumption of black tea/oolong tea had an increased OR for normal ALT levels after 12 months, with marginal significance (OR at ≥4 cups/ week, 2.40; 95% CI: 0.89-6.45).

**Table 4:** The association between consumption of selected beverages and normal ALT level (≤45 IU/L) during 12 months of follow-up among patients with normal ALT level at baseline (N=229).

Variable/level		After 6 months						After 12 months				
		Normal ALT		OR	95% CI	*P* value		Normal ALT		OR	95% CI	*P* value
		n/N	(%)					n/N	(%)			
Filtered coffee												
	Non-drinker	101/131	(77)	1				100/131	(76)	1		
	<1 cup/day	36/44	(82)	1.40	(0.54-3.61)	0.492		38/44	(86)	1.86	(0.65-5.27)	0.245
	≥1 cup/day	48/54	(89)	2.98	(1.06-8.34)	0.038		48/54	(89)	2.74	(1.00-7.54)	0.050
				(trend *P*=0.037)						(trend *P*=0.037)		
Unfiltered coffee												
	Non-drinker	98/122	(80)	1				99/122	(81)	1		
	<1 cup/day	32/38	(84)	1.36	(0.47-3.91)	0.568		33/38	(87)	1.78	(0.58-5.42)	0.311
	≥1 cup/day	55/69	(80)	1.33	(0.57-3.13)	0.513		54/69	(78)	1.22	(0.52-2.84)	0.651
				(trend *P*=0.491)						(trend *P*=0.593)		
Decaffeinated coffee												
	Non-drinker	178/219	(81)	1				180/219	(82)	1		
	Drinker	7/10	(70)	0.58	(0.12-2.93)	0.512		6/10	(60)	0.26	(0.06-1.15)	0.076
Green tea												
	≤1 cup/week	34/44	(77)	1				32/44	(73)	1		
	2 cups/week–1 cup/day	51/62	(82)	1.86	(0.63-5.51)	0.260		52/62	(84)	2.27	(0.78-6.59)	0.131
	≥2 cups/day	100/123	(81)	1.45	(0.57-3.72)	0.440		102/123	(83)	2.04	(0.81-5.11)	0.130
				(trend *P*=0.612)						(trend *P*=0.200)		
Black tea /Oolong tea												
	Non-drinker	57/75	(76)	1				58/75	(77)	1		
	<1 cup/week–3 cups/week	68/82	(83)	1.69	(0.71-4.00)	0.232		64/82	(78)	0.94	(0.41-2.17)	0.884
	≥4 cups/week	60/72	(83)	1.51	(0.62-3.71)	0.364		64/72	(89)	2.40	(0.89-6.45)	0.083
				(trend *P*=0.349)						(trend *P*=0.094)		

Model includes: sex, age (≤61, 62-70, 71+ years), body mass index (≤20, 21-23.4, 23.5+ kg/m2), smoking status (never/former, current), alcohol drinking status (never/former, current), diabetes mellitus, platelet count (<10 ×104/μL) and other beverages in the table.

When we further adjusted for baseline ALT level or duration of HCV infection, these results were almost unchanged (data not shown).

### Beverage consumption and ALT reduction among patients with higher baseline ALT

Among 147 patients, 63 patients (43%) and 61 patients (42%) showed ALT reduction of ≥10 IU/L after 6 months and 12 months, respectively. However, there were no significant associations with any beverages in the assessment using the logistic model.

When we investigated the association with ALT reduction of ≥20 IU/L, 34 patients (23%) and 39 patients (27%) had ALT reduction after 6 months and 12 months, respectively (Table 5). Logistic regression analysis showed that higher consumption of filtered coffee was significantly related to ALT reduction at each follow-up point. ORs (95% CI) of daily drinkers for ALT reduction were 4.88 (1.30-18.37) after 6 months and 3.79 (1.07-13.47) after 12 months, respectively. On the other hand, no decaffeinated coffee drinkers had ALT reduction. As for black tea/ oolong tea, patients with higher consumption showed significantly increased ORs for ALT reduction after 12 months (OR at ≥4 cups/week, 2.90; 95% CI: 1.00-8.37).

**Table 5:** The association between consumption of selected beverages and ALT reduction of 20 IU/L or more during 12 months of follow-up among patients with higher ALT level at baseline (N=147).

Variable/level		After 6 months						After 12 months				
		ALT reduction		OR	95%CI	*P* value		ALT reduction		OR	95%CI	*P* value
		n/N	(%)					n/N	(%)			
Filtered coffee												
	Non-drinker	17/89	(19)	1				20/89	(22)	1		
	<1 cup/day	8/28	(29)	3.69	(1.01-13.46)	0.048		8/28	(29)	2.07	(0.61-7.03)	0.245
	≥1 cup/day	9/30	(30)	4.88	(1.30-18.37)	0.019		11/30	(37)	3.79	(1.07-13.47)	0.040
				(trend *P*=0.012)						(trend *P*=0.034)		
Unfiltered coffee												
	Non-drinker	18/82	(22)	1				22/82	(27)	1		
	<1 cup/day	8/26	(31)	1.32	(0.36-4.78)	0.675		9/26	(35)	1.27	(0.35-4.56)	0.719
	≥1 cup/day	8/39	(21)	0.70	(0.22-2.22)	0.545		8/39	(21)	0.56	(0.18-1.73)	0.311
				(trend *P*=0.581)						(trend *P*=0.346)		
Decaffeinated coffee												
	Non-drinker	34/145	(23)					39/145	(27)			
	Drinker	0/2	(0)	-	-	-		0/2	(0)	-	-	-
Green tea												
	≤1 cup/week	14/45	(31)	1				15/45	(33)	1		
	2 cups/week–1 cup/day	6/38	(16)	0.28	(0.07-1.15)	0.077		10/38	(26)	0.53	(0.16-1.80)	0.306
	≥2 cups/day	14/64	(22)	0.72	(0.25-2.07)	0.536		14/64	(22)	0.51	(0.18-1.47)	0.212
				(trend *P*=0.660)						(trend *P*=0.228)		
Black tea /Oolong tea												
	Non-drinker	14/61	(23)	1				14/61	(23)	1		
	<1 cup/week–3 cups/week	8/41	(20)	0.89	(0.26-3.04)	0.857		6/41	(15)	0.85	(0.24-3.00)	0.803
	≥4 cups/week	12/45	(27)	1.04	(0.33-3.22)	0.952		19/45	(42)	2.90	(1.00-8.37)	0.050
				(trend *P*=0.950)						(trend *P*=0.036)		

Model includes: sex, age (≤61, 62-69, 70+ years), body mass index (≤20, 21-23, 24+ kg/m^2^), smoking status (never/former, current), alcohol drinking status (never/former, current), diabetes mellitus, platelet count (<10 ×10^4^/μL ) and other beverages in table except decaffeinated coffee.

In addition, we further evaluated the association with ALT reduction of ≥30 IU/L. Only filtered coffee consumption was significantly associated with ALT reduction. Daily consumption of filtered coffee increased the ORs for ALT reduction, both after 6 months (OR of daily drinkers, 10.48; 95% CI: 1.65-66.45) and after 12 months (OR of daily drinkers, 21.68; 95% CI: 1.69-278.63). However, a limited number of outcome events (18 patients after 6 months and 20 patients after 12 months, respectively) brought about the wider confidence intervals.

Table 6 summarizes the results of the logistic model for the association between baseline filtered coffee consumption and subsequent ALT reduction of ≥10 IU/L, ≥20 IU/L or ≥30 IU/L during 12 months. As the cutoff point of ALT reduction increased from 10 to 30 IU/L, increased ORs of daily drinkers were more pronounced, both after 6 months and after 12 months. It is noteworthy that patients who drank ≥1 cup/ day showed a 20-fold OR for ALT reduction of ≥30 IU/L after 12 months, although with a wider confidence interval.

**Table 6:** The association between filtered coffee consumption and ALT reduction of ≥10 IU/L, ≥20 IU/L or ≥30 IU/L during 12 months of follow-up among patients with higher ALT level at baseline (N=147).

		After 6 months						After 12 months				
Filtered coffee consumption		ALT reduction		OR	95% CI	*P* value		ALT reduction		OR	95% CI	*P* value
		n/N	(%)					n/N	(%)			
≥10 IU/L												
	Non-drinker	37/89	(42)	1				35/89	(39)	1		
	<1 cup/day	12/28	(43)	1.18	(0.42-3.26)	0.756		12/28	(43)	1.56	(0.57-4.28)	0.392
	≥1 cup/day	14/30	(47)	2.18	(0.77-6.14)	0.141		14/30	(47)	1.80	(0.64-5.05)	0.263
				(trend *P*=0.160)						(trend *P*=0.215)		
≥20 IU/L												
	Non-drinker	17/89	(19)	1				20/89	(22)	1		
	<1 cup/day	8/28	(29)	3.69	(1.01-13.46)	0.048		8/28	(29)	2.07	(0.61-7.03)	0.245
	≥1 cup/day	9/30	(30)	4.88	(1.30-18.37)	0.019		11/30	(37)	3.79	(1.07-13.47)	0.040
				(trend *P*=0.012)						(trend *P*=0.034)		
≥30 IU/L												
	Non-drinker	9/89	(10)	1				8/89	(9)	1		
	<1 cup/day	3/28	(11)	4.29	(0.55-33.26)	0.164		6/28	(21)	19.66	(2.08-185.96)	0.009
	≥1 cup/day	7/30	(23)	10.48	(1.65-66.45)	0.013		6/30	(20)	21.68	(1.69-278.63)	0.018
				(trend *P*=0.011)						(trend *P*=0.010)		

Model includes: sex, age (≤61, 62-69, 70+ years), body mass index (≤20, 21-23, 24+ kg/m^2^), smoking status (never/former, current), alcohol drinking status (never/former, current), diabetes mellitus, platelet count (<10 ×10^4^/μL), unfiltered coffee consumption, green tea consumption and black tea/ oolong tea consumption.

Even after adjusting for baseline ALT level or duration of HCV infection, these results were almost unchanged (data not shown).

## Discussion

In the present study among patients with chronic HCV infection, filtered coffee consumption exerted a consistently beneficial effect during 12 months of follow-up, not only for sustained normal ALT levels among patients with normal baseline ALT levels, but also for ALT reduction among patients with higher baseline ALT levels. A similar result was reported in the previous prospective study among Japanese office workers without liver dysfunction [21]. The present study is the first study in which coffee had a favorable effect also for ALT reduction of approximately 20-30 IU/L among liver disease patients with higher baseline ALT levels. The positive association between filtered coffee consumption and ALT reduction was likely to be stronger as the cutoff point of ALT reduction increased from 10 to 30 IU/L. Therefore, the effect of filtered coffee for ALT improvement seemed to be reliable. Although we could not show an effect for ALT reduction of 10 IU/L or more, the following interpretation could be considered. In patients with HCV infection, ALT levels fluctuate and may fall into normal range from time to time, as the natural course of the disease [26]. Thus, when outcome was regarded as an ALT reduction of ≥10 IU/L, the effect of coffee was likely to be masked by natural fluctuation.

According to previous reports, longer duration of HCV infection is positively associated with progression of liver fibrosis [27,28]. In addition, the baseline ALT level might confound the association between coffee consumption and subsequent ALT level. Therefore, we constructed a multivariate model in which the duration of HCV infection or baseline ALT level was additionally included as an explanatory variable. However, the results remained for the detection of a beneficial effect of filtered coffee consumption, suggesting that the effect of coffee on ALT level was free from the duration of HCV infection or baseline ALT level.

When the evidences on beneficial effect of coffee consumption for ALT have been accumulated, some might be more interested in the principal ingredients. A population-based cross-sectional study in United States showed the beneficial effects of coffee, especially caffeine consumption, in preventing elevated serum ALT level [20]. In addition, coffee and caffeine were negatively associated with serum ferritin [29], which might contribute to the mechanism of stabilizing ALT level. On the other hand, some studies indicated that diterpenes cafestol and kahweol in coffee, which are contained in a higher level in unfiltered boiled coffee compared with filtered coffee [30,31], might elevate the serum ALT level [30,32]. In the present study, coffee had a different effect on serum ALT level according to the brewing method: filtered coffee provided a beneficial effect, unfiltered coffee had no association, and decaffeinated coffee might have had a harmful effect on the stabilization of serum ALT level. These results seem to suggest that the key substances were included in filtered coffee. However, we could not perform further consideration on the specific ingredients in coffee because we did not obtain any information on the daily consumption of these suspected ingredients.

The relationship of green tea consumption on serum ALT level has been inconsistently reported in previous studies. Two studies in Japan showed that consumption of green tea did not materially influence the serum ALT level [15,19]. On the other hand, one study reported that heavy green tea consumption (≥10 cups per day) was related to decreased concentrations of serum ALT levels [33]. In the present study, no obvious association between green tea consumption and serum ALT level was obtained. However, our result might be criticized because of the following weakness. The tertiled categorization of the frequency of consumption was inappropriate for green tea, since about half of the patients were classified in the highest tertile category (≥2 cups/day). However, there was no association even when green tea consumption was classified into 4 categories (non-drinker, ≤1 cup/day, 2-3 cups/day and ≥4 cups/day). Thus, it might be more appropriate interpretation that green tea had no influence on serum ALT level during 12 months.

The results of the present study suggest that consumption of black tea/oolong tea has a beneficial effect on serum ALT level after 12 months, both among patients with normal baseline ALT levels and among those with higher ALT levels. To the best of our knowledge, this is the first study to report a favorable association between consumption of black tea/oolong tea and serum ALT level. However, we could not investigate the separate effects of black tea and oolong tea, since we collected the information as the combined consumption of black tea/oolong tea. Thus, to discuss the association in detail, further studies would be needed.

When interpreting the present results, the following limitations should be considered. The first limitation is an information bias in beverage consumption, because baseline beverage consumption was assessed solely at a single time point. Baseline consumption of caffeine-containing beverages might change according to the development of liver injury during the follow-up period. However, the progression to cirrhosis generally requires a long time [3]. Therefore, any change in habits with regard to caffeine-containing beverages because of the development of advanced liver disease is not conceivable during 12 months of follow-up. However, if caffeine-containing beverage consumption were changed by any other reason than liver disease progression, our results might be affected. The second limitation is that we did not ask about variations of cup size and coffee strength. Therefore, we cannot avoid some misclassification about the consumption of coffee and caffeine-containing beverages. However, it could be considered that the misclassification, if any, would have occurred equally in entire study subjects. Thus, the misclassification can be regarded as non-differential and does not materially affect the validity of the study results. The third limitation is that we could not evaluate the effect of unfiltered coffee separately into that of instant coffee, canned coffee and unfiltered boiled coffee, since we did not have the information about these unfiltered coffee products. In Japan, however, instant coffee and canned coffee are readily available as unfiltered coffee rather than unfiltered boiled coffee. Therefore, the present results of unfiltered coffee might represent the effects of instant coffee or canned coffee. Forth, regarding the confounders (i.e. body mass index, smoking status, alcohol drinking status and control of diabetes mellitus), we did not obtain any data on potential changes during the follow-up period. If some subjects had changed the status in these confounders during the follow-up period, this may affect the serum ALT levels. However, most of the study patients had been diagnosed as liver disease more than 10 years ago. Consequently, we could assume that these patients had been under the clinical follow-up for long time in the hospital. Thus, we considered that the potential changes during the study periods, if any, were limited. Finally, since we analyzed the data after excluding patients who started IFN therapy during the follow-up period and excluding patients with no follow-up data, some selection bias might have occurred. In fact, the excluded patients had higher baseline ALT levels than the analyzed patients, especially among the higher baseline ALT group, suggesting that the patients with higher ALT levels at baseline are more likely to have started IFN therapy during follow-up. In addition, the excluded patients were younger than the analyzed patients, both among the normal baseline ALT group and among the higher baseline ALT group. Therefore, it is not deniable that some bias in these factors might have affected the present results.

In conclusion, the present cohort study among patients with chronic HCV infection shows the beneficial effect of coffee consumption for stabilizing ALT level during 12 months of follow-up. The key substances seemed to be included in filtered coffee, because filtered coffee consistently showed a favorable effect on sustained normal ALT levels among patients with normal baseline ALT levels and on an ALT reduction of around 20-30 IU/L among patients with higher baseline ALT levels.
